# South Dakota

**Published:** 1897-04

**Authors:** 


### LEGISLATION.

*South Dakota.* A bill has been presented to the Legislature in this State looking to better regulations regarding the sheep industry and its protection from the dissemination of sheep-scab.

